# Weight trajectories and kidney function in early childhood

**DOI:** 10.1590/2175-8239-JBN-2026-0050en

**Published:** 2026-08-31

**Authors:** Margarida Dias, Sara Mosca, Laura Leite de Almeida, António Guerra, Ana Azevedo, Ana Cristina Santos, Franz Schaefer, Alberto Caldas Afonso, Liane Correia-Costa

**Affiliations:** 1 Universidade do Porto, Instituto de Ciências Biomédicas Abel Salazar, Porto, Portugal.; 2 Unidade Local de Saúde de Santo António, Centro Materno-Infantil do Norte, Departamento da Infância e Adolescência, Serviço de Nefrologia Pediátrica, Porto, Portugal.; 3 Universidade do Porto, Faculdade de Medicina, Departamento de Pediatria, Porto, Portugal.; 4 Universidade do Porto, Faculdade de Medicina, Departamento de Ciências da Saúde Pública e Forenses, e Educação Médica, Porto, Portugal.; 5 Universidade do Porto, Instituto de Saúde Pública, EPIUnit–ITR, Porto, Portugal.; 6 University of Heidelberg, Center for Pediatrics and Adolescent Medicine, Division of Pediatric Nephrology, Heidelberg, Germany.

**Keywords:** Body-Weight Trajectory, Childhood, Kidney Function Tests, Pediatric Obesity, Weight Gain

## Abstract

**Introduction:**

Postnatal growth profile is believed to have an impact on the risk of later obesity and of cardiovascular and kidney disease. We aimed to study the association between different trajectories of weight gain during infancy and early childhood and the renal function in prepubertal children.

**Methods:**

Longitudinal study of 1,004 children aged 7–9 years, divided into four groups of weight gain trajectories from birth to 6 years (I: eutrophic/reference; II: early-onset persistent accelerated weight gain; III: childhood-onset accelerated weight gain; and IV: early transient weight gain). Glomerular filtration rate (GFR) was estimated by several formulas using serum creatinine (Cr) and/or cystatin C (CysC), and by 24-hour creatinine clearance (CrCl).

**Results:**

Individuals in trajectory I presented the lowest Cr and CysC and the highest estimated GFR (eGFR) values, while trajectory II included those with the lowest eGFR, estimated by the Filler and Le Bricon formulas. Trajectory I individuals presented the lowest absolute CrCl values, while those in trajectory II presented the highest values. In multivariate linear regression models adjusted for age, sex, birth weight-for-gestational-age category, blood pressure, and current body mass index, trajectory III was associated with an eGFR that was 2.68 to 3.75 mL/min/1.73 m^2^ lower than that of trajectory I in the two models based on CysC.

**Conclusions:**

Children with early-onset persistent or childhood-onset accelerated weight gain presented significantly lower eGFR, which supports the influence of childhood growth patterns on later kidney function.

## INTRODUCTION

Postnatal growth is influenced by many factors and plays a major role in the development of children. Monitoring a child’s growth and development allows the identification of groups at greatest risk and in need of specific interventions, aiming to reduce infant morbidity, as well as to intervene in outcomes that these children may present later in life, improving chronic disease prevention. In fact, many studies have established that childhood growth patterns might have an important impact on the risk of later obesity and cardiovascular disease throughout life^1^.

Childhood obesity has become a global epidemic in the last decades and is well known to be associated with several adverse health outcomes in later childhood and adulthood. Overweight and obesity are now considered major public health problems. Rapid weight gain in the first 2 years of life, as well as excessive weight before puberty, has been shown to be associated with earlier pubertal maturation, both in boys and girls, and with a higher risk of obesity, diabetes, hypertension, and cardiovascular disease later in life^2^. A previous study found that early excessive weight gain is associated with thicker and stiffer arteries in childhood, reflecting a worse vascular profile^3^. Another study reported that accelerated weight gain during childhood and puberty was associated with a higher risk of coronary events in later adult life.

In recent years, evidence has suggested that the rise in the prevalence of obesity has paralleled the rise in the incidence of kidney disease^4^, indicating that obesity might be an independent risk factor for chronic kidney disease not only in adults but also in children^5^. In a previous study by our group, we reported that children with overweight and obesity aged 8 and 9 years already presented impaired renal function when compared to their non-overweight counterparts^6^. However, few studies have examined the impact of rapid weight gain, both in infancy and in childhood, on later kidney function.

In the present study, we aimed to evaluate the association between different trajectories of weight gain during infancy and early childhood and the renal function of prepubertal children. Several glomerular filtration rate (GFR) estimates based on creatinine and cystatin C values were compared among children aged 7–9 years, divided into 4 groups according to weight gain trajectories from birth to 6 years of age.

## METHODS

### Study design and sample

We studied children aged 7–9 years who have been followed since birth in a previously established birth cohort study in Porto, Portugal (Generation XXI)^7^. From the original cohort (n = 8,647), 4,590 children attended a face-to-face follow-up visit at 7 years of age, including anthropometric evaluation and blood sample collection, thus being eligible for the ObiKid project – a specific project aiming to clarify the impact of childhood obesity and associated comorbidities on the kidneys^6^. A group of 1,093 children (simple randomization using computer-generated random numbers) was preselected to have serum cystatin C measured (serum creatinine was already available for most of the children in the cohort); however, for the present study, we excluded 89 children: 50 children were excluded due to missing data on serum creatinine and 39 children due to the inability to estimate a weight trajectory (insufficient number of anthropometric measurements abstracted from previous records). Therefore, we finally included 1,004 participants in the present analysis. Among these participants, we assessed 24-hour creatinine clearance (CrCl) in 261 randomly selected children.

### Data collection and variable definition

The study visits were conducted at the Department of Public Health and Forensic Sciences and Medical Education at the *Faculdade de Medicina da Universidade do Porto*. Data on the children and their family medical history were collected by the application of a structured questionnaire, and perinatal information, such as gestational age and anthropometry at birth, had already been extracted from medical records in earlier evaluations. Children’s sex-specific birth weight-for-gestational-age categories were defined based on the revised Fenton growth charts^8^.

Anthropometric and general physical examinations were performed, including weight, height, and waist circumference. The body composition was assessed by foot-to-foot bioelectrical impedance analysis (Tanita^®^, model TBF-300). Body mass index (BMI) was calculated (kg/m^2^), and BMI-for-age was classified according to the World Health Organization (WHO) reference data for BMI z-score into the following categories: non-overweight (≤ +1 and ≥ -1 standard deviation, SD), overweight (> +1 SD and ≤ +2 SD) and obesity (> +2 SD)^9^. Blood pressure (BP) was measured in all children in the right arm using an aneroid sphygmomanometer (Elite 92125, Medel^®^, Italy), with a cuff size appropriate for the child’s arm circumference. BP measurements were taken three times with a 1-minute interval by a trained examiner, with the child in a seated position and the antecubital fossa supported at heart level, after at least a 5-minute rest. The second and third measurements were averaged for analysis.

### Laboratory procedures

Venous blood samples were collected after an overnight fast of at least 8 hours and analyzed for creatinine and cystatin C. All the laboratory analyses were performed in the Clinical Pathology Department of *Centro Hospitalar Universitário São João*, Porto, Portugal. The serum creatinine assay was based on the compensated Jaffé method, traceable to isotope dilution mass spectrometry (Olympus AU 5400 automated analyzer, Beckman-Coulter^®^, USA)^10^. Urinary creatinine was determined with the same clinical chemistry analyzer. Serum cystatin C was assayed using a particle-enhanced immunonephelometric assay (N Latex Cystatin C, Siemens^®^, Germany). To provide a broad assessment of renal function, several equations based on creatinine, cystatin C, or both biomarkers were applied. These equations were selected according to their availability and clinical use at the time the analytical plan was developed. To estimate GFR, in mL/min/1.73 m^2^, the following formulas were used: Revised Schwartz formula, GFR = k × (height/serum creatinine (Cr)); k = 0.413^11^; Filler formula, Log(GFR) = 1.962 + (1.123 × log(1/cystatin C (CysC)))^12^; Le Bricon formula, GFR = (78/CysC) + 4^13^; Combined Zappitelli formula, GFR = (507.76 × e^0.003 × height^)/(CysC^0.635^ × serum Cr^0.547^)^14^; Combined Schwartz formula, GFR = 39.8 × (height/serum Cr)^0.456^ × (1.8/CysC^0.418^ × (30/blood urea nitrogen)^0.079^ × (height/1.4)^0.179^ × (1.076 if female)^15^; Combined CKiD (Chronic Kidney Disease in Children) formula, GFR = 39.8 × (height/serum Cr)^0.456^ × (1.8/CysC)^0.418^ × (30/blood urea nitrogen)^0.079^ × (1.076 if male) or (1.00 if female) × (height/1.4)^0.17916^. Serum creatinine was expressed in mg/dL; cystatin C was expressed in mg/L; height was expressed in cm; age was expressed in years; blood urea nitrogen was expressed in mg/dL.

The 24-h CrCl (mL/min) was calculated according to the standard formula and considered the absolute CrCl. This value was then multiplied by 1.73 and divided by the child’s body surface area (BSA), determined by the Haycock formula^17^, to obtain the standardized CrCl (mL/min/1.73 m^2^), normalized to a body surface area of 1.73 m^2^. BSA using ideal body weight (IBW) (kg), instead of the child’s actual weight, was used as an alternative GFR adjuster. The IBW was inferred by calculating weight based on the 50^th^ percentile of BMI-for-age: IBW = BMI at the 50^th^ percentile (kg/m^2^) × height^2^ (m^2^)^18^.

Verbal and written information on the correct methods for 24-hour urine collection was provided to all children’s caretakers, and compliance was rechecked by a quick questionnaire upon sample delivery. In order to consider the 24-hour urine samples valid, urinary creatinine had to be between 11.3 and 28.0 mg/kg/day (according to age- and sex-specific reference values), and urinary volume had to be over 300 mL^19^.

### Weight trajectories definition

The determination of weight trajectories for participants in the Generation XXI study was based on an extensive dataset of anthropometric measurements extracted from the children’s health records, which were documented during routine care, from birth through age 6. For the final analysis and the definition of weight trajectories, some weight records were considered implausible and excluded (> 4 or < -4 SD from the mean), and all individuals with fewer than five weight measurements were also excluded. Furthermore, when more than two weight measurements were recorded within a month, the average of those measurements was used to attenuate autocorrelation. In the final estimation of weight trajectories,a total of 86,428 weight measurements (80.8% of all values extracted) from 5,237 children were considered, with a median of 16 weight measurement records (25^th^–75^th^ percentile [P25–P75]: 13–19) available per child. The weight trajectory patterns were defined by the intercept, slope, quadratic, and cubic random terms estimated by a mixed model (Normal Mixture Modeling for Model-Based Clustering). The most appropriate models were those that allowed the best homogeneous grouping of the individual growth patterns. This method for growth curve modeling has been described previously^20,21^.

**Figure 1:**
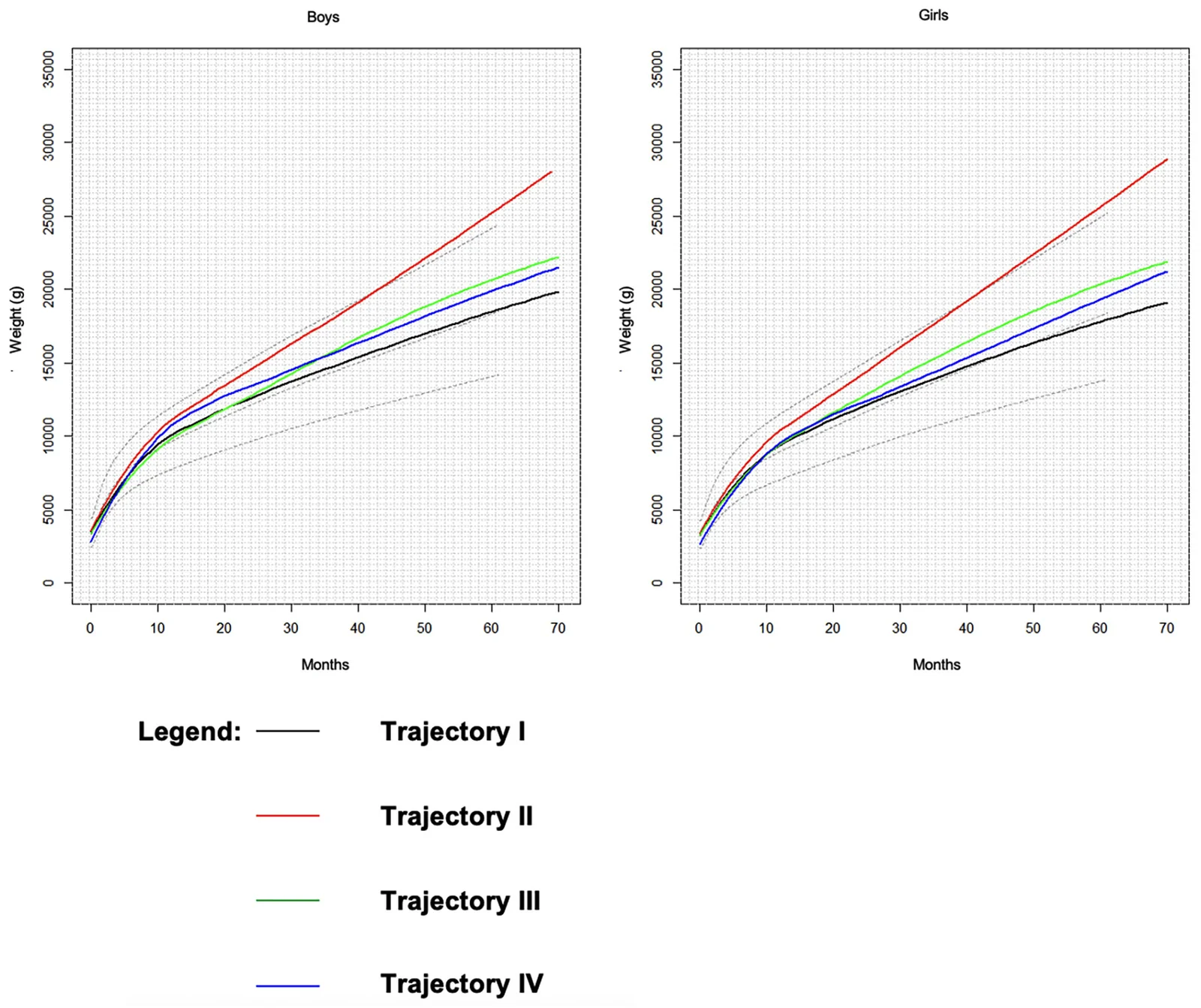
Weight trajectories defined in the Generation XXI cohort. The background gray lines represent the mean weight, the mean minus 2 standard deviations, and the mean plus 2 standard deviations of the World Health Organization reference population.

Finally, four different weight trajectories were defined, including children, regardless of sex, with similar growth patterns over time. For illustrative purposes and to relate the Generation XXI trajectories to universally used growth charts, the weight trajectories were plotted on the WHO growth charts (Figure 1). The four trajectories were labeled as “eutrophic/reference (normal weight gain)” (trajectory I), “early-onset persistent accelerated weight gain” (trajectory II), “childhood-onset accelerated weight gain” (trajectory III), and “early transient weight gain (accelerated weight gain during infancy)” (trajectory IV). For simplicity, we will henceforth refer to the periods of weight variation as infancy and childhood, the former including children aged less than 30 months (the first 2.5 years of life). Children in trajectory I exhibited a consistent course of weight gain, the slowest during the period analyzed, including children with the lowest body weight in our sample. Children in trajectory II diverged immediately after birth, showing rapid weight gain during the first 10 months of life. After this period, this trajectory exhibited a consistent weight gain pattern, the highest during the entire period of analysis, thus including the heaviest children. Trajectories I and III showed a similar weight gain pattern up to 20 months of age, but then trajectory III diverged, and the children showed a higher weight gain until the end of the analyzed period (constituting the second-heaviest group of children after 30 months). Finally, trajectory IV included the smallest babies at birth, who showed greater weight gain up to 10 months of age, corresponding to the trajectory with the second-heaviest group of children during this early infancy period. For purposes of analysis, all trajectories will be compared with trajectory I, considered the closest to the standard and desirable pattern of growth in childhood.

The 1,004 children in the current study sample were distributed across weight trajectories similarly to the remaining sample: 63.7%, 11.7%, 16.0%, and 8.6% versus 62.1%, 11.7%, 16.2%, and 10.0% in trajectories I, II, III, and IV, respectively (p = 0.551). At birth, they were slightly longer (49.0 versus 48.7 cm; p < 0.010) and heavier (3,236 versus 3,181 g; p = 0.010), possibly due to a slightly higher gestational age (38.8 versus 38.6 weeks; p < 0.010).

### Ethics

The ObiKid project was approved by the Ethics Committee of *Centro Hospitalar Universitário São João*, E.P.E., Porto, Portugal, and the *Faculdade de Medicina da Universidade do Porto*. It complies with the Helsinki Declaration, the guidelines for the ethical conduct of medical research involving children^22^, and the current national legislation. Written informed consent from parents or their legal guardians, as well as verbal assent from the children, was obtained for the collection of information and biological samples.

### Statistical analysis

Statistical analysis was performed using IBM^®^ SPSS^®^ Statistics 25.0. The one-way analysis of variance (ANOVA) test and chi-square tests were performed in order to analyze the differences in continuous and categorical variables between trajectories, respectively. Linear multivariate regression models were used to determine the effect of each trajectory on renal function markers and estimated glomerular filtration rate (eGFR); trajectory I was used as the reference. These models were adjusted for sex and children’s current age, birth weight-for-gestational-age category, systolic blood pressure (SBP) z-score, and BMI category (3 categories — non-overweight, overweight, and obesity). Data are presented as linear regression coefficients (β) and 95% confidence intervals (CI). All p values were two-sided and were considered statistically significant if p < 0.050.

## RESULTS

A total of 1,004 children (50.8% male) with a mean (SD) age of 7.5 (0.8) years were included in the present study.

General characteristics of the study sample by weight trajectories are presented in Table 1. At this age, 22.3% of the children were classified as overweight and 15.9% as having obesity. Among children with overweight/obesity, the mean (SD) BMI z-score was 1.53 (0.29) and 2.68 (0.49), respectively. There were several differences in both current and birth anthropometric measurements among trajectories, as depicted in Table 1. Trajectory I included children with the lowest mean values of weight z-score, BMI z-score, and SBP z-score, as well as the highest gestational age; the highest proportion of children with normal weight was found in this trajectory. Conversely, trajectory II was associated with the highest mean values of weight z-score, BMI z-score, SBP z-score, diastolic blood pressure (DBP) z-score, and birth weight. The second-highest mean values of weight z-score and BMI z-score were found in trajectory III. Trajectory IV presented the lowest mean values of DBP z-score.

**Table 1:** Baseline characteristics and current anthropometric and blood pressure data, by weight trajectories.

	Weight trajectory					
	All n = 1,004	I n = 640	II n = 117	III n = 161	IV n = 86	p-value
Demographic and birth data						
Age (months)	89.99 (9.62)	89.76 (9.50)	89.21 (9.05)	90.78 (10.15)	91.24 (10.18)	0.297
Male sex	510 (50.8%)	347 (54.2%)	64 (54.7%)	51 (31.7%)	48 (55.8%)	<0.001
Gestational age (weeks)	38.82 (1.53)	39.01 (1.19)	38.97 (1.26)	38.95 (1.26)	37.03 (2.85)	<0.001
Birth weight (g)	3,232.7 (463.4)	3,262.1 (393.7)	3,474.4 (461.5)	3,190.7 (374.3)	2,768.2 (710.8)	<0.001
Birth weight-for-gestational-age category^a^						
SGA	70 (7.0%)	41 (6.4%)	4 (3.4%)	13 (8.1%)	12 (14.0%)	<0.001
AGA	881 (87.9%)	570 (89.3%)	96 (82.1%)	145 (90.1%)	70 (81.4%)	
LGA	51 (5.1%)	27 (4.2%)	17 (14.5%)	3 (1.9%)	4 (4.7%)	
Anthropometric data						
Weight (kg)	28.05 (6.69)	25.70 (4.93)	35.64 (7.59)	30.88 (6.60)	29.97 (6.68)	<0.001
z-score	0.79 (1.17)	0.33 (0.94)	2.28 (1.07)	1.36 (0.88)	1.15 (0.99)	<0.001
Height (cm)	126.32 (6.99)	124.67 (6.52)	130.98 (7.00)	128.32 (6.45)	128.52 (6.93)	<0.001
z-score	0.38 (0.95)	0.09 (0.86)	1.28 (0.95)	0.69 (0.80)	0.66 (0.83)	<0.001
BMI (kg/m^2^)	17.39 (2.74)	16.42 (1.98)	20.61 (3.12)	18.62 (2.79)	17.96 (2.59)	<0.001
z-score	0.78 (1.19)	0.36 (1.00)	2.14 (1.08)	1.31 (1.05)	1.06 (1.08)	<0.001
BMI category^b^						
Non-overweight	620 (61.8%)	485 (75.8%)	23 (19.7%)	68 (42.2%)	44 (51.2%)	<0.001
Overweight	224 (22.3%)	121 (18.9%)	28 (23.9%)	49 (30.4%)	26 (30.2%)	
Obesity	160 (15.9%)	34 (5.3%)	66 (54.4%)	44 (27.3%)	16 (18.6%)	
Office SBP (mmHg)	104 (9)	103 (9)	108 (9)	106 (9)	104 (7)	<0.001
z-score	0.02 (0.90)	-0.13 (0.88)	0.52 (0.83)	0.24 (0.86)	0.02 (0.88)	<0.001
Office DBP (mmHg)	67 (8)	67 (7)	69 (9)	68 (8)	65 (8)	0.011
z-score	0.52 (0.68)	0.45 (0.65)	0.85 (0.75)	0.61 (0.67)	0.40 (0.69)	<0.001

Abbreviations – SGA, small for gestational age; AGA, adequate for gestational age; LGA, large for gestational age; BMI, body mass index; SBP, systolic blood pressure; DBP, diastolic blood pressure.

Notes – Values are presented as means (standard deviations) or numbers (percentages), as appropriate.

^a^Birth weight-for-gestational-age categories were defined according to the Fenton growth charts^8^.

^b^BMI z-scores categories were defined according to the World Health Organization classification^9^.

Table 2 presents renal function markers and eGFR values by weight trajectories. Differences were observed among the distinct trajectories in mean serum creatinine and cystatin C levels, eGFR estimated by the Filler and Le Bricon equations, and mean absolute CrCl levels. Trajectory I included the individuals with the lowest serum creatinine and cystatin C levels and the highest eGFR, whereas trajectory II included those with the lowest estimates: 150 (SD 18) versus 145 (SD 15) mL/min/1.73 m^2^ according to the Filler equation (p = 0.002) and 125 (SD 13) versus 122 (SD 11) mL/min/1.73 m^2^ according to the Le Bricon equation (p = 0.002). Trajectory I included the individuals with the lowest absolute CrCl levels, while trajectory II included those with the highest: 98 (SD 24) versus 115 (SD 23) mL/min (p = 0.005). In addition, children in trajectory I presented the highest BSA-adjusted 24-h CrCl, whereas those in trajectory II presented the lowest; however, the differences were not statistically significant. The opposite was observed when CrCl was adjusted for BSA-IBW, with trajectory I including children with the lowest BSA-IBW-adjusted CrCl and trajectory II including those with the highest, but again, the difference was not statistically significant. BSA-IBW-adjusted CrCl values were lower in trajectory I (compared with trajectories II, III, and IV) but higher than those observed for BSA-adjusted 24-h CrCl in the same group.

**Table 2:** Renal function markers and estimated glomerular filtration rates of the study sample by weight trajectories.

	All n = 1,004	Weight trajectory				p-value
		I n = 640	II n = 117	III n = 161	IV n = 86	
Renal function markers						
Serum creatinine (mg/dL)	0.41 (0.06)	0.41 (0.06)	0.42 (0.06)	0.42 (0.06)	0.42 (0.06)	0.024
Serum cystatin C (mg/L)	0.66 (0.07)	0.65 (0.07)	0.67 (0.06)	0.67 (0.07)	0.67 (0.07)	0.003
GFR estimates						
GFR (Cr_Schwartz) (mL/min/1.73 m^2^)	129 (18)	129 (18)	131 (17)	129 (18)	128 (16)	0.437
GFR (CysC_Filler) (mL/min/1.73 m^2^)	149 (18)	150 (18)	145 (15)	146 (16)	147 (18)	0.002
GFR (CysC_Le Bricon) (mL/min/1.73 m^2^)	124 (13)	125 (13)	122 (11)	122 (12)	123 (13)	0.002
GFR (Cr-CysC_Zappitelli) (mL/min/1.73 m^2^)	138 (16)	139 (17)	137 (15)	136 (16)	136 (15)	0.107
GFR (Cr-CysC_Schwartz) (mL/min/1.73 m^2^)	111 (12)	110 (12)	111 (11)	112 (12)	110 (10)	0.323
GFR (Cr-CysC_CKiD) (mL/min/1.73 m^2^)	111 (11)	112 (11)	112 (12)	109 (11)	111 (10)	0.089
Absolute 24-h CrCl* (mL/min)	101 (24)	98 (24)	115 (23)	104 (21)	105 (24)	0.005
BSA-adjusted 24-h CrCl* (mL/min/1.73 m^2^)	160 (33)	162 (35)	154 (24)	155 (30)	155 (32)	0.341
BSA-IBW-adjusted CrCl* (mL/min/1.73 m^2^)	171 (36)	169 (37)	182 (30)	174 (34)	173 (36)	0.370

Abbreviations – GFR, glomerular filtration rate; Cr, creatinine; CysC, cystatin C; CKiD, Chronic Kidney Disease in Children; CrCl, 24-h creatinine clearance; BSA, body surface area calculated using the Haycock equation; IBW, ideal body weight; BSA-IBW, body surface area calculated using the Haycock equation with ideal body weight instead of the child’s actual weight.

Notes – Values are presented as means (standard deviations).

*In the analysis of 24-h CrCl, only 261 children with valid 24-h urine samples were included (165 in trajectory I, 25 in trajectory II, 45 in trajectory III, and 26 in trajectory IV).

In multivariate linear regression models (Table 3), adjusted for the children’s sex and current age, birth weight-for-gestational-age category, SBP z-score, and BMI category (3 categories – non-overweight, overweight, and obesity), trajectory III was associated with an eGFR that was 2.68 to 3.75 mL/min/1.73 m^2^ lower, in the two models based on the cystatin C Le Bricon and Filler equations, respectively, in comparison to trajectory I.

**Table 3:** Mean differences in renal function markers and estimated glomerular filtration rates across the different weight trajectories, using trajectory I as the reference.

GFR estimates	Weight trajectory			
	I* β (95%CI)	II β (95%CI)	III β (95%CI)	IV β (95%CI)
GFR (Cr_Schwartz) (mL/min/1.73 m^2^)	0	1.48 (-2.54; 5.51)	-0.04 (-3.34; 3.27)	-0.97 (-5.06; 3.12)
GFR (CysC_Filler) (mL/min/1.73 m^2^)	0	-2.87 (-6.78; 1.04)	-3.75 (-6.95; -0.54)***	-2.63 (-6.61; 1.34)
GFR (CysC_Le Bricon) (mL/min/1.73 m^2^)	0	-2.05 (-4.86; 0.76)	-2.68 (-4.98; -0.38)***	-1.89 (-4.75; 0.96)
GFR (Cr-CysC_Zappitelli) (mL/min/1.73 m^2^)	0	-1.45 (-5.13; 2.22)	-2.51 (-5.52; 0.51)	-2.46 (-6.20; 1.27)
GFR (Cr-CysC_Schwartz) (mL/min/1.73 m^2^)	0	0.54 (-1.86; 2.93)	-0.53 (-2.49; 1.44)	-0.90 (-3.34; 1.53)
GFR (Cr-CysC_CKiD) (mL/min/1.73 m^2^)	0	0.59 (-1.81; 2.99)	-0.60 (-2.56; 1.37)	-0.91 (-3.35; 1.52)
Absolute 24-h CrCl** (mL/min)	0	3.93 (-6.21; 14.07)	0.33 (-7.73; 8.39)	-1.10 (-10.34; 8.15)
BSA-adjusted 24-h CrCl** (mL/min/1.73 m^2^)	0	-7.67 (-23.12; 7.78)	-3.63 (-15.91; 8.66)	-7.95 (-22.03; 6.14)
BSA-IBW-adjusted CrCl** (mL/min/1.73 m^2^)	0	-4.87 (-21.04; 11.29)	-2.41 (-15.26; 10.44)	-6.83 (-21.56; 7.91)

Abbreviations – GFR, glomerular filtration rate; Cr, creatinine; CysC, cystatin C; CKiD, Chronic Kidney Disease in Children; CrCl, 24-h creatinine clearance; BSA, body surface area calculated using the Haycock equation; IBW, ideal body weight; BSA-IBW, body surface area calculated using the Haycock equation with ideal body weight instead of the child’s actual weight.

Notes – The values presented are linear regression coefficients (β) and 95% confidence intervals, estimated by multivariate linear regression models. The models were adjusted for current age (months), sex, birth weight-for-gestational-age category (according to the Fenton growth charts^8^), systolic blood pressure z-score, and BMI category (3 categories – non-overweight, overweight, and obesity).

*Reference trajectory.

**In the analysis of 24-h CrCl only 261 children with valid 24-h urine samples were included (165 in trajectory I, 25 in trajectory II, 45 in trajectory III, and 26 in trajectory IV).

***p < 0.050.

## DISCUSSION

In the present study, we found that both creatinine and cystatin C were lower in children with slower and consistent weight gain (trajectory I), whilst eGFR was higher in these children. In contrast, children with early-onset persistent or childhood-onset accelerated weight gain (trajectories II and III) presented lower eGFR values. Additionally, prepubertal children with excessive weight gain during childhood (trajectory III) showed significantly lower eGFR estimated by the Filler and Le Bricon equations, regardless of the children’s current age and sex, birth weight-for-gestational-age category, and BMI category. We also found that, in a subsample of children, absolute CrCl values were lower in the group with consistent weight gain (trajectory I), whereas the highest values were found among those with early-onset persistent excessive weight gain (trajectory II).

The relationship between weight trajectories and kidney function during childhood has not yet been established. In fact, there are no previous studies addressing this specific issue. However, some studies have already associated specific growth trajectories with adverse cardiovascular and metabolic outcomes in later childhood^2,23^ and adulthood^2^.

Regarding the association between obesity and renal damage, there is now strong evidence in adults that obesity is associated with a substantial increase in the incidence of chronic kidney disease^5^ and, in recent years, more and more studies have tried to ascertain whether such an association exists in children^24^. However, no studies have examined the impact of rapid weight gain, either during infancy or childhood, on later kidney function. Many authors have reported inverse correlations between obesity or related anthropometric indexes and GFR in children^25,26^, including a previous study from our group^6^. Nonetheless, there are still conflicting results in the literature. Some studies have failed to demonstrate statistically significant differences in GFR values between children with obesity and their normal-weight counterparts, others found no statistically significant differences after adjustment for metabolic factors^4^, and others reported higher GFR values in children with obesity, possibly due to an initial phase of hyperfiltration known to occur in the pathophysiology of obesity-related kidney damage. In the present study, we found that children in weight trajectories associated with increased weight gain presented significantly lower eGFR values (according to some equations) but higher absolute CrCl values.

The initial changes in renal function described in the context of obesity are glomerular hyperperfusion, hyperfiltration, followed by hypertension and progressive renal function decline. At this age, we would expect to find higher eGFR values in children with obesity, which would be consistent with an initial phase of renal damage, but, as discussed in a previous study by our group, the use of classic GFR formulas adjusted for BSA may introduce a bias, given that BMI has a strong correlation with BSA^27^. Therefore, in this context, GFR values adjusted for BSA are considerably underestimated in individuals with higher BMI, since an overadjustment is introduced into GFR estimation^28^. This overadjustment might falsely mask the initial phase of glomerular hyperfiltration^29,30^. IBW-based BSA has been identified as a promising option for GFR adjustment since it has recently been found to avoid overcorrection and GFR underestimation, both in adult and pediatric studies, and has already been recognized as useful in other clinical areas, such as indexing ventricular mass in children^31^. In fact, a previous study by our group found that BSA-IBW-adjusted GFR values were significantly higher in children with overweight/obesity (as opposed to what happened when comparing BSA-adjusted GFR values, which were lower in children with overweight/obesity) and showed a better correlation with absolute GFR values^27^. Our results seem to be in line with these previous findings, since children in weight trajectories associated with obesity, especially those with persistent excessive weight gain, presented lower values of BSA-adjusted CrCl (154 (24) mL/min/1.73 m^2^), but higher values of BSA-IBW-adjusted CrCl (182 (30) mL/min/1.73 m^2^), although these differences were not statistically significant. It is possible that this sustained difference over time leads to kidney damage. Regarding CrCl, the lack of significant results may be related to the low statistical power, since few children were included in this analysis.

Many recent studies have addressed the issue of defining an accurate method for estimating GFR in children, based on either creatinine or cystatin C, given that most of the widely used equations were primarily developed in children with reduced GFR and may lead to underestimation in individuals without renal damage^32^. In addition, body composition is known to also influence the accuracy of eGFR equations. In adults with obesity, higher cystatin C levels have been previously described in patients with higher BMI, leading to underestimation when compared with exact exogenous GFR determinations^33^. Several studies have highlighted the importance of cystatin C as a filtration marker for GFR estimation in children^34,35^. However, other studies have shown that the use of combined equations should be preferred, particularly in groups in which body composition may play a role^36^, since, in children, estimates based on cystatin C may also yield a significant underestimation in those with higher BMI^6^. In our study, trajectories with excessive weight gain were associated with lower eGFR values, especially when estimated using cystatin C-based equations. Thus, these findings may be partially explained by the limitations of cystatin C discussed in the context of obesity. Nonetheless, in the multivariate models, the lower eGFR values estimated by the two cystatin C-based equations in trajectory III persisted after adjustment for current BMI. This reinforces that, even considering the intrinsic limitations of the available estimation methods, children with excessive weight gain during childhood already present impaired renal function at the age of 7–9 years.

Furthermore, we reported that children in weight trajectories associated with obesity, particularly those with persistent excessive weight gain, presented higher absolute CrCl values compared with those with slower and consistent weight gain. The increased absolute values found in the trajectories with greater weight gain might be indicative that these children could in fact be in an initial phase of hyperfiltration caused by higher blood pressure associated with excessive body mass. The analysis of these results increases our ability to draw more definitive conclusions about the direction of the association found between weight gain and GFR. However, regardless of the direction of the aforementioned association, our findings probably represent the first evidence of the early impact of accelerated weight gain, both in infancy and in childhood, on glomerular function in young children. In this context, our findings of lower eGFR and higher absolute CrCl values in children with increased weight gain can be explained by the fact that GFR estimated by both creatinine- and cystatin C-based equations and normalized to 1.73 m^2^ of body surface area might underestimate GFR in children with overweight/obesity, in comparison with the absolute CrCl values, which do not take body size into account.

In addition, birth weight and gestational age may play an important role in GFR later in life. Since the number of nephrons is fixed at birth, children with intrauterine growth restriction, born small for gestational age or preterm and, consequently, with reduced nephron endowment, not only have an increased risk of obesity but are also more susceptible to developing chronic kidney disease in the future. Excessive weight gain, particularly during rapid catch-up growth, promotes an increase in the metabolic and hemodynamic load of each individual nephron, leading to a stage of hyperfiltration, which is even more evident in individuals with reduced nephron mass^37^. In the present study, linear regression models used to evaluate the effect of each trajectory on eGFR values were adjusted for birth weight-for-gestational-age categories, confirming that the observed differences in GFR were independent of this potential confounding factor.

The major strength of our study is the inclusion of a large sample of healthy prepubertal children, which is expected to be representative of the population in question, leading us to believe that our findings can be generalized. In fact, we present a prospective analysis of data from a population-based cohort, from early life through mid-childhood, showing a previously undescribed association. The multiple weight measurements obtained from birth allowed a longitudinal analysis of the impact of weight gain on renal function, considering its “duration”, “intensity”, and age of onset. Moreover, we used growth mixture modeling, which is considered an ideally flexible modeling approach to identify subpopulations with similar longitudinal trajectories. The analysis of our outcome one year after the last exposure measurement seems appropriate for a longitudinal approach. Additionally, we performed cystatin C and creatinine measurements in a large subsample of the cohort—comprising more than 1,000 children aged 7–9 years—allowing GFR estimation based on both markers, an approach that, as previously discussed, has advantages over single-marker-based equations. The availability of detailed information regarding numerous pre- and postnatal variables enabled us to consider the impact of potential confounders on the associations studied.

Nonetheless, some important limitations of our study should be acknowledged. The analysis of renal function in a cross-sectional manner demands caution when interpreting causality. We intend to overcome this limitation by continuing to perform regular evaluations of these children, with particular interest in their nutritional status and the evolution of their renal function. Another important limitation is that we could only assess renal function by 24-hour urinary CrCl in 261 of the 1,004 children enrolled in the study. This reinforces the need for future data collection to better ascertain the nature of the association between weight gain trajectories and renal function, not only during childhood but also later in adulthood, and to evaluate the possibility of an increased incidence of chronic kidney disease and poorer renal survival among adults who, as children, presented a worse growth profile. Another weakness of our study is that we considered only a general measure (weight) and not fat distribution or other biochemical markers. Weight trajectories were chosen because repeated weight measurements were consistently available from birth through early childhood, allowing for robust longitudinal modeling. Nevertheless, weight does not distinguish between fat mass and lean mass, nor does it reflect body fat distribution. Therefore, some trajectories may partly capture constitutional somatic growth in addition to adiposity. Future studies using direct body-composition measures or other biochemical markers may allow a more detailed analysis and help refine these associations. However, we believe that this potential limitation was largely mitigated by the large amount of longitudinal data available, with thousands of weight records over time, which would be impossible to obtain for other biomarkers.

Although statistically significant, the differences in eGFR between groups were modest in absolute terms, and values generally remained within expected pediatric reference ranges. Therefore, these findings should not be interpreted as overt renal dysfunction but rather as possible early physiologic or subclinical differences that warrant longitudinal follow-up. The coexistence of lower indexed eGFR estimates and higher absolute CrCl values in higher-weight trajectories may reflect methodological issues related to body-size normalization, early hyperfiltration, or both. Additional kidney injury markers were not available and would have strengthened the overall interpretation.

Our study has led us to conclude that children with early-onset persistent or childhood-onset accelerated weight gain presented significantly lower eGFR values when compared with children with slower and consistent weight gain. These findings may reflect early hemodynamic or methodological differences rather than established kidney dysfunction. Our results support the influence of growth patterns on kidney function, which may be in line with other previously reported associations between excessive weight gain trajectories and a higher risk of developing obesity and cardiovascular risk factors later in life^38^. Since the children included in our study are very young, still presenting renal function in the normal range, further work needs to be carried out to establish the clinical relevance of our findings. As the onset and development of obesity-associated renal disease are subtle, often remaining undetected for years, the widespread use of early markers that have already shown good results as screening methods for early renal damage in children with obesity^4^ might play an important role in the future by improving the early detection and prevention of renal damage in this setting.

Our findings highlight the importance of preventing obesity from early life. Future investigations focusing on the influence of growth patterns on cardiovascular and renal outcomes are crucial to help the early identification of at-risk children and to implement timely interventions to improve lifelong health.

## Data Availability

The datasets generated and/or analyzed during the current study are available from the corresponding author upon reasonable request.
