# Supplementary material for: Weight trajectories and kidney function in early childhood
Source: J Bras Nefrol. 2026 Aug 31;48(4):e20260050. doi: 10.1590/2175-8239-JBN-2026-0050en (PMC13537207; doi:10.1590/2175-8239-JBN-2026-0050en)
Supplement: Translation

# Translation: Trajetórias de peso e função renal na infância

**DOI:** 10.1590/2175-8239-JBN-2026-0050pt

**Authors:** Margarida Dias, Sara Mosca, Laura Leite de Almeida, António Guerra, Ana Azevedo, Ana Cristina Santos, Franz Schaefer, Alberto Caldas Afonso, Liane Correia-Costa

**Affiliations:** 1 Universidade do Porto, Instituto de Ciências Biomédicas Abel Salazar, Porto, Portugal.; 2 Unidade Local de Saúde de Santo António, Centro Materno-Infantil do Norte, Departamento da Infância e Adolescência, Serviço de Nefrologia Pediátrica, Porto, Portugal.; 3 Universidade do Porto, Faculdade de Medicina, Departamento de Pediatria, Porto, Portugal.; 4 Universidade do Porto, Faculdade de Medicina, Departamento de Ciências da Saúde Pública e Forenses, e Educação Médica, Porto, Portugal.; 5 Universidade do Porto, Instituto de Saúde Pública, EPIUnit–ITR, Porto, Portugal.; 6 University of Heidelberg, Center for Pediatrics and Adolescent Medicine, Division of Pediatric Nephrology, Heidelberg, Germany.

**Keywords:** Aumento de Peso, Infância, Obesidade Pediátrica, Testes de Função Renal, Trajetória do Peso do Corpo

## Abstract

**Introdução:**

O perfil de crescimento pós-natal tem impacto no risco de obesidade tardia e de doenças cardiovasculares e renais. O objetivo do presente estudo foi avaliar a associação entre diferentes trajetórias de ganho de peso durante a infância precoce e tardia e a função renal em crianças pré-púberes.

**Métodos:**

Estudo longitudinal com 1.004 crianças com idades entre 7 e 9 anos, divididas em 4 grupos de trajetórias de ganho de peso desde o nascimento até os 6 anos (I: eutrófico/referência; II: ganho de peso acelerado persistente de início precoce; III: ganho de peso acelerado de início na infância; e IV: ganho de peso transitório precoce). A taxa de filtração glomerular (TFG) foi estimada por várias fórmulas, utilizando creatinina sérica (Cr) e/ou cistatina C (CisC), bem como pelo clearance de creatinina em 24 horas (CrCl).

**Resultados:**

Indivíduos na trajetória I apresentaram os valores mais baixos de Cr e CisC e os valores mais elevados de TFG estimada, enquanto a trajetória II incluiu aqueles com a TFG mais baixa, estimada pelas fórmulas de Filler e Le Bricon. Os indivíduos da trajetória I apresentaram os valores absolutos mais baixos de CrCl, enquanto os da trajetória II apresentaram os valores mais elevados. Nos modelos de regressão linear multivariada, ajustados para idade, sexo, classe de peso ao nascimento, pressão arterial e índice de massa corporal atual, a trajetória III esteve associada a uma TFG estimada 2,68 a 3,75 mL/min/1,73 m^2^ menor que a da trajetória I nos dois modelos baseados na CisC.

**Conclusões:**

As crianças com ganho de peso persistente de início precoce ou com ganho de peso acelerado de início na infância apresentaram uma TFG estimada significativamente mais baixa, o que corrobora a influência dos padrões de crescimento infantis na função renal posterior.

## INTRODUÇÃO

O crescimento pós-natal é influenciado por diversos fatores e desempenha um papel fundamental no desenvolvimento das crianças. O monitoramento do crescimento e do desenvolvimento de uma criança permite-nos identificar os grupos de maior risco e que necessitam de intervenções específicas, com o objetivo de reduzir a morbidade infantil, bem como de intervir sobre os desfechos que essas crianças poderão apresentar futuramente, melhorando a prevenção de doenças crônicas. De fato, muitos estudos demonstraram que os padrões de crescimento na infância podem ter um impacto importante no risco de obesidade e de doenças cardiovasculares ao longo da vida^1^.

A obesidade infantil tornou-se uma epidemia global nas últimas décadas, com associações bem conhecidas com vários desfechos adversos para a saúde na infância tardia e na idade adulta. O excesso de peso ou a obesidade são agora considerados um grave problema de saúde pública. Foi demonstrado que o rápido ganho de peso nos primeiros 2 anos de vida, bem como o excesso de peso antes da puberdade, estão associados à maturação puberal precoce, tanto no sexo masculino quanto no sexo feminino, e a um risco mais elevado de obesidade, diabetes, hipertensão e doenças cardiovasculares em fases posteriores da vida^2^. Um estudo prévio demonstrou que o ganho de peso excessivo precoce está associado a artérias mais espessas e rígidas na infância, refletindo um perfil vascular mais desfavorável^3^. Outro estudo relatou que o ganho de peso acelerado durante a infância e a puberdade está associado a um risco mais elevado de eventos coronários na vida adulta.

Nos últimos anos, evidências têm demonstrado que o aumento da prevalência de obesidade tem acompanhado o aumento da incidência de doença renal^4^, o que sugere que a obesidade pode ser um fator de risco independente para a doença renal crônica, não só em adultos, mas também em crianças^5^. Em um estudo anterior realizado por nosso grupo, relatamos que crianças com excesso de peso e obesidade, com idades entre 8 e 9 anos, já apresentavam alterações na função renal quando comparadas a seus pares sem excesso de peso^6^. No entanto, poucos estudos avaliaram o impacto do rápido ganho de peso, tanto na infância precoce quanto na infância tardia, na função renal em fases posteriores da vida.

No presente estudo, pretendeu-se analisar a associação entre diferentes trajetórias de ganho de peso durante a infância precoce e tardia, bem como a função renal em crianças pré-púberes. Foram comparadas várias estimativas da taxa de filtração glomerular (TFG) com base nos valores de creatinina e cistatina C, em crianças com idades entre 7 e 9 anos, divididas em quatro grupos de trajetórias de ganho de peso desde o nascimento até os 6 anos de idade.

## MÉTODOS

### Desenho do estudo e amostra

O estudo analisou crianças com idades entre 7 e 9 anos, acompanhadas desde o nascimento no âmbito de um estudo de *coorte* de nascimentos previamente estabelecido no Porto, Portugal (Geração XXI)^7^. Da *coorte* original (n = 8.647), 4.590 crianças compareceram a uma consulta presencial de acompanhamento aos 7 anos de idade, que incluiu avaliação antropométrica e coleta de amostras de sangue, tornando-se, assim, elegíveis para o projeto ObiKid – um projeto específico que visa esclarecer o impacto da obesidade infantil e das comorbidades associadas nos rins^6^. Um grupo de 1.093 crianças (randomização simples utilizando números aleatórios gerados por computador) foi pré-selecionado para a medição da cistatina C sérica (a creatinina sérica já estava disponível para a maioria das crianças da *coorte*); entretanto, para o presente estudo, excluímos 89 crianças: 50 foram excluídas devido à falta de dados sobre a creatinina sérica e 39, devido à impossibilidade de estimar uma trajetória de peso (número insuficiente de medidas antropométricas extraídas de registros anteriores). Assim, incluímos por fim, 1.004 participantes na presente análise. Destes, avaliamos o *clearance* de creatinina (CrCl) em 24 horas em 261 crianças selecionadas aleatoriamente.

### Coleta de dados e definição das variáveis

As consultas do estudo ocorreram no Departamento de Ciências da Saúde Pública e Forenses, e Educação Médica da Faculdade de Medicina da Universidade do Porto. Os dados sobre as crianças e o histórico médico familiar foram coletados por meio da aplicação de um questionário estruturado, e as informações perinatais, tais como idade gestacional e antropometria ao nascimento, já haviam sido extraídas dos registros médicos em avaliações anteriores. As classes de peso ao nascer específicas por sexo para a idade gestacional foram definidas com base nas curvas de crescimento de Fenton revisadas^8^.

Foram realizados exames antropométricos e exames físicos gerais, incluindo peso, estatura e perímetro abdominal, e a composição corporal foi avaliada por análise de impedância bioelétrica pé-pé (Tanita^®^, modelo TBF-300). O índice de massa corporal (IMC) foi calculado (kg/m^2^) e o IMC para a idade foi classificado de acordo com os dados de referência da Organização Mundial da Saúde (OMS) para o *z-score* do IMC nas seguintes categorias: sem excesso de peso (≤ +1 e ≥ −1 desvio padrão [DP]), excesso de peso (> +1 DP e ≤ +2 DP) e obesidade (> +2 DP)^9^. A pressão arterial (PA) foi medida em todas as crianças no braço direito utilizando um esfigmomanômetro aneroide (Elite 92125, Medel^®^, Itália), com um manguito de tamanho adequado à circunferência do braço da criança. As medições da PA foram realizadas três vezes, com intervalo de 1 minuto, por um examinador treinado, com a criança na posição sentada e a fossa antecubital apoiada na altura do coração, após repouso de, pelo menos, 5 minutos. Utilizou-se para análise a média da segunda e da terceira medições.

### Procedimentos laboratoriais

Foram coletadas amostras de sangue venoso após um jejum noturno de, pelo menos, 8 horas, as quais foram analisadas com o objetivo de determinar os níveis de creatinina e cistatina C. Todas as análises laboratoriais foram realizadas no Serviço de Patologia Clínica do Centro Hospitalar Universitário São João, no Porto, Portugal. A determinação da creatinina sérica baseou-se no método de Jaffé compensado, rastreável à espectrometria de massa por diluição isotópica (analisador automatizado Olympus AU 5400, Beckman-Coulter^®^, EUA)^10^. A creatinina urinária foi determinada com o mesmo analisador de química clínica. A cistatina C sérica foi analisada por meio da técnica de imunonefelometria com reforço de partículas (*N Latex Cystatin C*, Siemens^®^, Alemanha). Para realizar uma avaliação abrangente da função renal, foram aplicadas diferentes equações baseadas na creatinina, na cistatina C ou em ambos os biomarcadores. Essas equações foram selecionadas de acordo com sua disponibilidade e utilização clínica à época em que a análise foi desenvolvida. Para estimar a TFG, em mL/min/1,73 m^2^, foram utilizadas as seguintes fórmulas: fórmula de Schwartz revisada, TFG = k × (estatura/creatinina sérica (Cr)); k = 0,413^11^; fórmula de Filler, Log(TFG) = 1,962 + (1,123 × log(1/cistatina C (CisC)))^12^; fórmula de Le Bricon, TFG = (78/CisC) + 4^13^; fórmula combinada de Zappitelli, TFG = (507,76 × e^0,003 × estatura^)/(CisC^0,635^ × Cr sérica^0,547^)^14^; fórmula combinada de Schwartz, TFG = 39,8 × (estatura/Cr sérica)^0,456^ × (1,8/CisC^0,418^ × (30/nitrogênio ureico no sangue)^0,079^ × (estatura/1,4)^0,179^ × (1,076 para o sexo feminino)^15^; fórmula combinada CKiD (*Chronic Kidney Disease in Children*), TFG = 39,8 × (estatura/Cr sérica)^0,456^ × (1,8/CisC)^0,418^ × (30/nitrogênio ureico no sangue)^0,079^ × (1,076 para o sexo masculino ou 1,00 para o sexo feminino) × (estatura/1,4)^0,17916^. A creatinina sérica foi expressa em mg/dL; a cistatina C foi expressa em mg/L; a estatura foi expressa em cm; a idade foi expressa em anos; o nitrogênio ureico no sangue foi expresso em mg/dL.

O CrCl de 24 horas (mL/min) foi calculado de acordo com a fórmula padrão e considerado como o CrCl absoluto, que foi então multiplicado por 1,73 e dividido pela área de superfície corporal (ASC) da criança (determinada pela fórmula de Haycock^17^), a fim de obter o valor padrão do CrCl em mL/min/1.73 m^2^ (normalizado para 1,73 m^2^ de superfície corporal). A ASC utilizando o peso corporal ideal (PCI) (kg), em alternativa ao peso real da criança, foi utilizada como um ajustador alternativo da TFG. O PCI foi calculado com base no percentil 50 do IMC para a idade: PCI = IMC no percentil 50 (kg/m^2^) × estatura^2^ (m^2^)^18^.

Foram fornecidas informações orais e escritas sobre os métodos corretos de coleta de urina de 24 horas a todos os responsáveis pelas crianças e a adesão às orientações foi novamente verificada por meio de um questionário rápido no momento da entrega das amostras. Para que as amostras de urina de 24 horas fossem consideradas válidas, a creatinina urinária deveria situar-se entre 11,3 e 28,0 mg/kg/dia (de acordo com os valores de referência específicos para a idade e o sexo), e o volume urinário deveria ser superior a 300 mL^19^.

### Definição das trajetórias de peso

A definição das trajetórias de peso dos participantes do estudo Geração XXI baseou-se em um vasto conjunto de dados de medições antropométricas extraídas dos boletins de saúde das crianças, registradas no âmbito dos cuidados de rotina, desde o nascimento até os 6 anos de idade. Para a análise final e a definição das trajetórias de peso, alguns registros de peso foram considerados implausíveis e excluídos (desvios padrão > 4 ou < -4 em relação à média) e todos os indivíduos com menos de cinco medições foram igualmente excluídos. Além disso, quando foram registradas mais de duas avaliações de peso em um mês, considerou-se a média dessas medições para atenuar a autocorrelação. Na estimativa final das trajetórias de peso, foram consideradas um total de 86.428 medições de peso (80,8% de todos os valores extraídos) de 5.237 crianças, com mediana de 16 (percentis 25 e 75 [P25–P75]: 13–19) registros de medições de peso disponíveis por criança. Os padrões das trajetórias de peso foram estimados por um modelo misto (*Normal Mixture Modeling for Model-Based Clustering*). Os modelos mais adequados foram aqueles que permitiram o melhor agrupamento homogêneo dos padrões individuais de crescimento. Esse método de modelagem de curvas de crescimento já foi descrito anteriormente^20,21^.

Por fim, foram definidas quatro trajetórias de peso diferentes, que incluíram crianças, independentemente do sexo, com padrões de crescimento semelhantes ao longo do tempo. Para fins ilustrativos e para relacionar as trajetórias do Geração XXI às tabelas de crescimento universalmente utilizadas, as trajetórias de peso foram representadas graficamente nas curvas de crescimento da OMS (Figura 1). As quatro trajetórias foram designadas como “eutrófico/referência (ganho de peso normal)” (trajetória I), “ganho de peso acelerado persistente de início precoce” (trajetória II), “ganho de peso acelerado de início na infância” (trajetória III) e “ganho de peso transitório precoce (ganho de peso acelerado durante a primeira infância)” (trajetória IV). Por uma questão de simplicidade, passaremos a referir-nos aos períodos de variação de peso como primeira infância e infância, incluindo no primeiro período as crianças com menos de 30 meses de idade (os primeiros 2,5 anos de vida). As crianças na trajetória I apresentaram uma evolução consistente do ganho de peso, a mais lenta durante o período analisado, incluindo aquelas com o menor peso da nossa amostra. As crianças na trajetória II divergiram imediatamente após o nascimento, apresentando um rápido ganho de peso durante os primeiros 10 meses de vida. Após esse período, essa trajetória apresentou um padrão de ganho de peso consistente, o mais elevado durante todo o período de análise, incluindo, assim, as crianças mais pesadas. As trajetórias I e III apresentaram um padrão de ganho de peso semelhante até os 20 meses de idade. No entanto, a partir daí, a trajetória III divergiu, e as crianças apresentaram ganho de peso mais acentuado até o final do período analisado (constituindo o segundo grupo de crianças mais pesadas após os 30 meses). Por fim, a trajetória IV incluiu os recém-nascidos menores ao nascimento, com ganho de peso mais elevado até os 10 meses de idade, correspondendo à trajetória com o segundo grupo de crianças mais pesadas nesse período da primeira infância. Para fins de análise, todas as trajetórias serão comparadas com a trajetória I, considerada a mais próxima do padrão de crescimento ideal e desejável na infância.

**Figura 1:** Trajetórias de peso definidas na *coorte* Geração XXI. As linhas cinzentas de fundo representam o peso médio, a média menos 2 desvios padrão e a média mais 2 desvios padrão da população de referência da Organização Mundial de Saúde.

As 1.004 crianças da amostra do presente estudo distribuíram-se pelas trajetórias de peso de forma semelhante à da amostra restante: 63,7%, 11,7%, 16,0% e 8,6% versus 62,1%, 11,7%, 16,2% e 10,0% nas trajetórias I, II, III e IV, respectivamente (p = 0,551). Ao nascimento, eram ligeiramente mais compridas (49,0 versus 48,7 cm; p < 0,010) e mais pesadas (3.236 versus 3.181 g; p = 0,010), possivelmente devido a uma idade gestacional ligeiramente superior (38,8 versus 38,6 semanas; p < 0,010).

### Ética

O projeto ObiKid foi aprovado pela Comissão de Ética do Centro Hospitalar Universitário São João, E.P.E., Porto, Portugal, e pela Faculdade de Medicina da Universidade do Porto. Encontra-se em conformidade com a Declaração de Helsinque, as diretrizes para a conduta ética da pesquisa médica envolvendo crianças^22^ e a legislação nacional em vigor. Foi obtido o consentimento informado por escrito dos pais ou de representantes legais e o assentimento verbal das crianças, no que diz respeito à coleta de informações e amostras biológicas.

### Análise estatística

A análise estatística foi realizada utilizando o software IBM^®^ SPSS^®^ *Statistics* versão 25.0. Foram realizados o teste de análise de variância unidirecional (ANOVA) e os testes de qui-quadrado, a fim de analisar as diferenças nas variáveis contínuas e categóricas entre as trajetórias, respectivamente. Modelos de regressão linear multivariada foram utilizados para determinar o efeito de cada trajetória nos marcadores de função renal e na TFG estimada; a trajetória I foi utilizada como referência. Os modelos foram ajustados para sexo e idade atual das crianças, classe de peso ao nascimento para a idade gestacional, *z-score* da pressão arterial sistólica (PAS) e categoria de IMC (3 categorias – sem excesso de peso, excesso de peso e obesidade). Os dados são apresentados como coeficientes de regressão linear (β) e intervalos de confiança (IC) de 95%. Todos os valores de p são bilaterais, sendo considerados estatisticamente significativos se p < 0,050.

## RESULTADOS

Um total de 1.004 crianças (50,8% do sexo masculino) com uma idade média (DP) de 7,5 (0,8) anos foi incluído no presente estudo.

As características gerais da amostra do estudo por trajetórias de peso são apresentadas na Tabela 1. Nesta idade, 22,3% foram classificados como crianças com excesso de peso e 15,9% como crianças com obesidade. Entre aquelas com excesso de peso/obesidade, o *z-score* do IMC médio (DP) foi de 1,53 (0,29) e 2,68 (0,49), respectivamente. Verificaram-se diferenças nos dados antropométricos atuais e ao nascimento entre as trajetórias, conforme ilustrado na Tabela 1. A trajetória I incluiu crianças com os valores médios mais baixos do *z-score* de peso, do *z-score* de IMC e do *z-score* de PAS, e a idade gestacional mais elevada; a maior proporção de indivíduos com peso normal foi encontrada nessa trajetória. Por outro lado, a trajetória II foi associada aos valores médios mais elevados do *z-score* de peso, do *z-score* de IMC, do *z-score* de PAS, do *z-score* de pressão arterial diastólica (PAD) e do peso ao nascimento. Os segundos maiores valores médios do *z-score* de peso e do *z-score* de IMC foram encontrados na trajetória III. A trajetória IV apresentou os valores médios mais baixos do *z-score* de PAD.

**Tabela 1:** Características gerais e dados antropométricos e de pressão arterial atuais, por trajetórias de peso.

	Total n = 1.004	Trajetórias de peso				Valor de p
		I n = 640	II n = 117	III n = 161	IV n = 86	
Dados demográficos e de nascimento						
Idade (meses)	89,99 (9,62)	89,76 (9,50)	89,21 (9,05)	90,78 (10,15)	91,24 (10,18)	0,297
Sexo masculino	510 (50,8%)	347 (54,2%)	64 (54,7%)	51 (31,7%)	48 (55,8%)	<0,001
Idade gestacional (semanas)	38,82 (1,53)	39,01 (1,19)	38,97 (1,26)	38,95 (1,26)	37,03 (2,85)	<0,001
Peso ao nascer (g)	3.232,7 (463,4)	3.262,1 (393,7)	3.474,4 (461,5)	3.190,7 (374,3)	2.768,2 (710,8)	<0,001
Peso ao nascer para a idade gestacional^a^						
PIG	70 (7,0%)	41 (6,4%)	4 (3,4%)	13 (8,1%)	12 (14,0%)	<0,001
AIG	881 (87,9%)	570 (89,3%)	96 (82,1%)	145 (90,1%)	70 (81,4%)	
GIG	51 (5,1%)	27 (4,2%)	17 (14,5%)	3 (1,9%)	4 (4,7%)	
Dados antropométricos						
Peso (kg)	28,05 (6,69)	25,70 (4,93)	35,64 (7,59)	30,88 (6,60)	29,97 (6,68)	<0,001
*z-score*	0,79 (1,17)	0,33 (0,94)	2,28 (1,07)	1,36 (0,88)	1,15 (0,99)	<0,001
Estatura (cm)	126,32 (6,99)	124,67 (6,52)	130,98 (7,00)	128,32 (6,45)	128,52 (6,93)	<0,001
z-score	0,38 (0,95)	0,09 (0,86)	1,28 (0,95)	0,69 (0,80)	0,66 (0,83)	<0,001
IMC (kg/m^2^)	17,39 (2,74)	16,42 (1,98)	20,61 (3,12)	18,62 (2,79)	17,96 (2,59)	<0,001
*z-score*	0,78 (1,19)	0,36 (1,00)	2,14 (1,08)	1,31 (1,05)	1,06 (1,08)	<0,001
Categorias de IMC^b^						
Sem excesso de peso	620 (61,8%)	485 (75,8%)	23 (19,7%)	68 (42,2%)	44 (51,2%)	<0,001
Excesso de peso	224 (22,3%)	121 (18,9%)	28 (23,9%)	49 (30,4%)	26 (30,2%)	
Obesidade	160 (15,9%)	34 (5,3%)	66 (54,4%)	44 (27,3%)	16 (18,6%)	
PAS no consultório (mmHg)	104 (9)	103 (9)	108 (9)	106 (9)	104 (7)	<0,001
*z-score*	0,02 (0,90)	-0,13 (0,88)	0,52 (0,83)	0,24 (0,86)	0,02 (0,88)	<0,001
PAD no consultório (mmHg)	67 (8)	67 (7)	69 (9)	68 (8)	65 (8)	0,011
*z-score*	0,52 (0,68)	0,45 (0,65)	0,85 (0,75)	0,61 (0,67)	0,40 (0,69)	<0,001

Abreviaturas – AIG, adequado para a idade gestacional; GIG, grande para a idade gestacional; IMC, índice de massa corporal; PAD, pressão arterial diastólica; PAS, pressão arterial sistólica; PIG, pequeno para a idade gestacional.

Notas – Os valores são apresentados como média (desvio padrão) ou número (%), conforme apropriado.

^a^As classes de peso ao nascimento em relação à idade gestacional foram definidas de acordo com as curvas de crescimento de Fenton^8^.

^b^As categorias de z-scores do IMC foram definidas de acordo com a classificação da Organização Mundial da Saúde^9^.

A Tabela 2 apresenta os marcadores da função renal e os valores da TFG estimada por trajetórias de peso. Foram observadas diferenças entre as distintas trajetórias quanto aos níveis médios de creatinina sérica e cistatina C, à TFG (estimada pelas fórmulas de Filler e Le Bricon) e aos níveis médios de CrCl absoluto. A trajetória I incluiu os indivíduos com os menores níveis de creatinina sérica e cistatina C e a TFG estimada mais elevada, enquanto a trajetória II incluiu aqueles com os valores mais baixos de TFG estimada: segundo Filler, 150 (DP 18) *vs.* 145 (DP 15) mL/min/1,73 m^2^ (p = 0,002) e, segundo Le Bricon, 125 (DP 13) *vs.* 122 (DP 11) mL/min/1,73 m^2^ (p = 0,002). A trajetória I incluiu as crianças com os menores níveis de CrCl absoluto, enquanto a trajetória II incluiu aquelas com os mais elevados: 98 (DP 24) *vs.* 115 (DP 23) mL/min (p = 0,005). Além disso, as crianças na trajetória I apresentaram os valores mais elevados de CrCl de 24 horas ajustado pela ASC, enquanto as da trajetória II apresentaram os valores mais baixos, mas as diferenças não foram estatisticamente significativas. Observou-se o oposto quando o CrCl foi ajustado pela ASC-PCI, com a trajetória I incluindo crianças com os menores valores de CrCl ajustado pela ASC-PCI e a trajetória II incluindo aquelas com os mais elevados; porém, novamente, a diferença não foi estatisticamente significativa. Os valores de CrCl ajustados pela ASC-PCI foram mais baixos na trajetória I (quando comparados às trajetórias II, III e IV), mas mais elevados do que os encontrados para o CrCl de 24 horas ajustado pela ASC no mesmo grupo.

**Tabela 2:** Marcadores de função renal e taxas de filtração glomerular estimadas da amostra do estudo, por trajetórias de peso.

	Total n = 1.004	Trajetórias de peso				Valor de p
		I n = 640	II n = 117	III n = 161	IV n = 86	
Marcadores da função renal						
Creatinina sérica (mg/dL)	0,41 (0,06)	0,41 (0,06)	0,42 (0,06)	0,42 (0,06)	0,42 (0,06)	0,024
Cistatina C sérica (mg/L)	0,66 (0,07)	0,65 (0,07)	0,67 (0,06)	0,67 (0,07)	0,67 (0,07)	0,003
Estimativas da TFG						
TFG (Cr_Schwartz) (mL/min/1,73 m^2^)	129 (18)	129 (18)	131 (17)	129 (18)	128 (16)	0,437
TFG (CisC_Filler) (mL/min/1,73 m^2^)	149 (18)	150 (18)	145 (15)	146 (16)	147 (18)	0,002
TFG (CisC_Le Bricon) (mL/min/1,73 m^2^)	124 (13)	125 (13)	122 (11)	122 (12)	123 (13)	0,002
TFG (Cr-CisC_Zappitelli) (mL/min/1,73 m^2^)	138 (16)	139 (17)	137 (15)	136 (16)	136 (15)	0,107
TFG (Cr-CisC_Schwartz) (mL/min/1,73 m^2^)	111 (12)	110 (12)	111 (11)	112 (12)	110 (10)	0,323
TFG (Cr-CisC_CKiD) (mL/min/1,73 m^2^)	111 (11)	112 (11)	112 (12)	109 (11)	111 (10)	0,089
CrCl absoluto em 24 horas* (mL/min)	101 (24)	98 (24)	115 (23)	104 (21)	105 (24)	0,005
CrCl em 24 horas ajustado pela ASC* (mL/min/1,73 m^2^)	160 (33)	162 (35)	154 (24)	155 (30)	155 (32)	0,341
CrCl em 24 horas ajustado pela ASC-PCI* (mL/min/1,73 m^2^)	171 (36)	169 (37)	182 (30)	174 (34)	173 (36)	0,370

Abreviaturas – ASC, área de superfície corporal calculada pela equação de Haycock; ASC-PCI, área de superfície corporal calculada pela equação de Haycock utilizando o peso corporal ideal em vez do peso corporal real da criança; CisC, cistatina C; CKiD, *Chronic Kidney Disease in Children*; Cr, creatinina; CrCl, clearance de creatinina em 24 horas; PCI, peso corporal ideal; TFG, taxa de filtração glomerular.

Notas – Os valores são apresentados como média (desvio padrão).

*Na análise do CrCl de 24 horas, foram incluídas apenas 261 crianças com amostras válidas de urina de 24 horas (165 na trajetória I, 25 na trajetória II, 45 na trajetória III e 26 na trajetória IV).

Nos modelos de regressão linear multivariada (Tabela 3), ajustados para sexo e idade atual das crianças, classe de peso ao nascimento para a idade gestacional, *z-score* da PAS e categoria de IMC (3 categorias – sem excesso de peso, excesso de peso e obesidade), a trajetória III foi associada a uma TFG estimada 2,68 a 3,75 mL/min/1,73 m^2^ inferior, nos dois modelos baseados nas fórmulas de Le Bricon e Filler utilizando cistatina C, respectivamente, em comparação com a trajetória I.

**Tabela 3:** Diferenças médias dos marcadores de função renal e nas taxas de filtração glomerular estimadas nas diferentes trajetórias de peso, utilizando a trajetória I como referência.

Estimativas da TFG	Trajetórias de peso			
	I* β (IC95%)	II β (IC95%)	III β (IC95%)	IV β (IC95%)
TFG (Cr_Schwartz) (mL/min/1,73 m^2^)	0	1,48 (-2,54; 5,51)	-0,04 (-3,34; 3,27)	-0,97 (-5,06; 3,12)
TFG (CisC_Filler) (mL/min/1,73 m^2^)	0	-2,87 (-6,78; 1,04)	-3,75 (-6,95; -0,54)***	-2,63 (-6,61; 1,34)
TFG (CisC_Le Bricon) (mL/min/1,73 m^2^)	0	-2,05 (-4,86; 0,76)	-2,68 (-4,98; -0,38)***	-1,89 (-4,75; 0,96)
TFG (Cr-CisC_Zappitelli) (mL/min/1,73 m^2^)	0	-1,45 (-5,13; 2,22)	-2,51 (-5,52; 0,51)	-2,46 (-6,20; 1,27)
TFG (Cr-CisC_Schwartz) (mL/min/1,73 m^2^)	0	0,54 (-1,86; 2,93)	-0,53 (-2,49; 1,44)	-0,90 (-3,34; 1,53)
TFG (Cr-CisC_CKiD)(mL/min/1,73 m^2^)	0	0,59 (-1,81; 2,99)	-0,60 (-2,56; 1,37)	-0,91 (-3,35; 1,52)
CrCl absoluto em 24 horas** (mL/min)	0	3,93 (-6,21; 14,07)	0,33 (-7,73; 8,39)	-1,10 (-10,34; 8,15)
CrCl em 24 horas ajustado pela ASC** (mL/min/1,73 m^2^)	0	-7,67 (-23,12; 7,78)	-3,63 (-15,91; 8,66)	-7,95 (-22,03; 6,14)
CrCl em 24 horas ajustado pela ASC-PCI** (mL/min/1,73 m^2^)	0	-4,87 (-21,04; 11,29)	-2,41 (-15,26; 10,44)	-6,83 (-21,56; 7,91)

Abreviaturas – ASC, área de superfície corporal calculada pela equação de Haycock; ASC-PCI, área de superfície corporal calculada pela equação de Haycock utilizando o peso corporal ideal em vez do peso corporal real da criança; CisC, cistatina C; CKiD, *Chronic Kidney Disease in Children*; Cr, creatinina; CrCl, clearance de creatinina em 24 horas; PCI, peso corporal ideal; TFG, taxa de filtração glomerular.

Notas – Os valores apresentados são coeficientes de regressão linear (β) e intervalos de confiança de 95%, estimados por modelos de regressão linear multivariada. Os modelos foram ajustados para a idade atual das crianças em meses, sexo, classe de peso ao nascer em relação à idade gestacional (de acordo com as curvas de crescimento de Fenton 8), *z-score* da pressão arterial sistólica e categoria de IMC das crianças (3 categorias – sem excesso de peso, excesso de peso e obesidade).

*Trajetória de referência.

**Na análise do CrCl de 24 horas, foram incluídas apenas 261 crianças com amostras válidas de urina de 24 horas (165 na trajetória I, 25 na trajetória II, 45 na trajetória III e 26 na trajetória IV).

***O valor de p é < 0,050.

## DISCUSSÃO

No presente estudo, constatamos que tanto os valores de creatinina quanto os de cistatina C eram mais baixos em crianças com um ganho de peso mais lento e consistente (trajetória I), enquanto a TFG estimada era mais elevada nessas crianças. Em contrapartida, os indivíduos com ganho de peso persistente de início precoce ou acelerado de início na infância (trajetórias II e III) apresentaram valores de TFG estimada mais baixos. Além disso, as crianças pré-púberes com ganho de peso excessivo durante a infância (trajetória III) apresentaram valores significativamente mais baixos de TFG estimados pelas fórmulas de Filler e Le Bricon, independentemente de idade atual e sexo, classe de peso ao nascimento para a idade gestacional e categoria de IMC. Verificamos também que, em uma subamostra de crianças, os valores absolutos de CrCl eram inferiores no grupo com ganho de peso consistente (trajetória I), enquanto os valores mais elevados foram encontrados entre aqueles com ganho de peso excessivo persistente de início precoce (trajetória II).

A relação entre as trajetórias de peso e a função renal durante a infância ainda não foi estabelecida. De fato, não existem estudos anteriores que abordem diretamente essa questão. No entanto, alguns estudos já associaram trajetórias específicas de crescimento a desfechos cardiovasculares e metabólicos adversos na infância tardia^2,23^ e na idade adulta^2^.

No que diz respeito à associação entre obesidade e lesão renal, existem atualmente fortes evidências em adultos de que a obesidade está associada a um aumento significativo da incidência de doença renal crônica^5^. Nos últimos anos, cada vez mais estudos têm procurado determinar se tal associação também existe em crianças^24^. No entanto, nenhum estudo avaliou o impacto do rápido ganho de peso, tanto na primeira infância quanto na infância, na função renal subsequente. Muitos autores relataram correlações inversas entre obesidade ou índices antropométricos relacionados e a TFG em crianças^25,26^, incluindo um estudo anterior realizado pelo nosso grupo^6^. Ainda assim, existem resultados contraditórios na literatura. Alguns estudos não conseguiram demonstrar diferenças estatisticamente significativas nos valores de TFG entre crianças com obesidade e seus pares com peso normal; outros não alcançaram essa diferença após ajuste para fatores metabólicos^4^; e há ainda estudos que descreveram valores mais elevados de TFG em crianças com obesidade, possivelmente devido a uma fase inicial de hiperfiltração, conhecida por ocorrer na fisiopatologia da lesão renal relacionada à obesidade. No presente trabalho, relatamos que crianças pertencentes a trajetórias associadas a um maior ganho de peso apresentaram TFG estimada significativamente mais baixa (de acordo com algumas fórmulas), mas um CrCl absoluto mais elevado.

As alterações iniciais da função renal descritas no contexto da obesidade são: hiperperfusão glomerular, hiperfiltração e, posteriormente, hipertensão e declínio progressivo da função renal. Nessa faixa etária, seria de se esperar encontrar valores mais elevados de TFG estimada em crianças com obesidade, compatíveis com uma fase inicial de lesão renal; no entanto, conforme discutido em um estudo anterior de nosso grupo, o uso de fórmulas clássicas de TFG ajustadas para ASC pode introduzir um viés, considerando-se o fato de que o IMC apresenta forte correlação com a ASC^27^. Nesse contexto, os valores de TFG ajustados pela ASC são consideravelmente subestimados em indivíduos com IMC mais elevado, uma vez que introduz um sobreajuste na estimativa da TFG^28^. Esse sobreajuste pode mascarar falsamente a fase inicial da hiperfiltração glomerular^29,30^. A ASC baseada no PCI foi identificada como uma opção promissora para o ajuste da TFG. Estudos recentes demonstraram que essa abordagem evita a correção excessiva e a subestimativa da TFG, tanto em adultos quanto na população pediátrica. Além disso, essa estratégia já foi reconhecida como útil em outras áreas clínicas, como na indexação da massa ventricular em crianças^31^. De fato, um estudo anterior de nosso grupo constatou que os valores da TFG ajustados pela ASC-PCI eram significativamente mais elevados em crianças com sobrepeso/obesidade (em oposição ao que foi observado com os valores da TFG ajustados pela ASC, os quais eram inferiores nessas crianças) e apresentavam melhor correlação com os valores absolutos da TFG^27^. Nossos resultados parecem estar em consonância com esses achados anteriores, uma vez que os participantes pertencentes a trajetórias de peso associadas à obesidade, especialmente aqueles com ganho de peso excessivo persistente, apresentaram valores mais baixos de CrCl ajustado pela ASC (154 (24) mL/min/1,73 m^2^), mas valores mais elevados de CrCl ajustado pela ASC-PCI (182 (30) mL/min/1,73 m^2^), embora sem significância estatística. É possível que essa diferença sustentada ao longo do tempo conduza à lesão renal. No que diz respeito ao CrCl, a ausência de resultados significativos pode estar relacionada ao baixo poder estatístico da análise, considerando o número reduzido de crianças que foram incluídas.

Muitos estudos recentes abordaram a questão da definição de um método preciso para estimar a TFG em crianças, seja com base na creatinina ou na cistatina C, uma vez que a maioria das equações amplamente utilizadas foi desenvolvida principalmente em crianças com TFG reduzida e pode levar à subestimativa da TFG em indivíduos sem lesão renal^32^. Além disso, sabe-se que a composição corporal também pode influenciar a precisão dessas equações. Em adultos com obesidade, níveis mais altos de cistatina C já foram descritos em pacientes com IMC mais elevado, o que conduz a uma subestimativa dos valores da TFG quando comparados às determinações exógenas exatas dessa medida^33^. Diversos estudos destacaram a importância da cistatina C como marcador de filtração para estimar a TFG em crianças^34,35^. Por outro lado, outros estudos demonstraram que o uso de equações combinadas deve ser preferido, particularmente em grupos nos quais a composição corporal possa desempenhar um papel^36^, uma vez que, na população pediátrica, as estimativas baseadas exclusivamente na cistatina C também podem resultar em uma subestimativa significativa em crianças com IMC mais elevado^6^. Em nosso estudo, as trajetórias com ganho de peso excessivo foram associadas a valores mais baixos de TFG, especialmente quando esta foi estimada por equações baseadas na cistatina C. Assim, é possível levantar a hipótese de que esses achados sejam parcialmente explicados pelas limitações desse marcador discutidas no contexto da obesidade. No entanto, nos modelos multivariados, os valores reduzidos de TFGe, estimados pelas duas equações baseadas na cistatina C na trajetória III persistiram após o ajuste para o IMC atual. Esse resultado reforça que, mesmo considerando as limitações intrínsecas dos métodos de estimativa, as crianças com ganho de peso excessivo durante a infância já apresentam função renal alterada entre os 7 e 9 anos de idade.

Além disso, relatamos que as crianças pertencentes a trajetórias de peso associadas à obesidade, particularmente aquelas com ganho de peso excessivo persistente, apresentavam valores absolutos de CrCl mais elevados em comparação com aquelas com ganho de peso mais lento e consistente. Os valores absolutos aumentados encontrados nas trajetórias com maior ganho de peso podem ser indicativos de que essas crianças poderiam, de fato, estar em uma fase inicial de hiperfiltração causada pela pressão arterial mais elevada associada ao excesso de massa corporal. A análise desses resultados amplia nossa capacidade de extrair conclusões mais definitivas sobre a direção da associação encontrada entre o ganho de peso e a TFG. No entanto, independentemente da direção da associação acima referida, nossos resultados provavelmente representam a primeira evidência do impacto precoce do ganho de peso acelerado, tanto na primeira infância quanto na infância, na função glomerular de crianças pequenas. Nesse contexto, nossos achados de TFGe mais baixa e CrCl absoluto mais elevado em crianças com ganho de peso excessivo podem ser explicados pelo fato de que a TFG estimada tanto pelas equações de creatinina quanto pelas de cistatina C, e normalizada para 1,73 m^2^ de superfície corporal, pode subestimar a TFG em crianças com sobrepeso/obesidade, em comparação com os valores absolutos do CrCl, que não levam em consideração o tamanho corporal.

Adicionalmente, o peso ao nascer e a idade gestacional podem desempenhar um papel importante na TFG em fases posteriores da vida. Uma vez que o número de néfrons é fixo ao nascimento, crianças com restrição de crescimento intrauterino, nascidas pequenas para a idade gestacional ou prematuras e, por conseguinte, com um número reduzido de néfrons, não só apresentam um risco aumentado de obesidade, como também são mais suscetíveis a desenvolver doença renal crônica no futuro. O ganho excessivo de peso, particularmente durante o rápido crescimento de recuperação, promove um aumento da carga metabólica e hemodinâmica de cada néfron individual, levando a um estágio de hiperfiltração, o que é ainda mais evidente em indivíduos com número reduzido de néfrons^37^. No presente estudo, os modelos de regressão linear para avaliar o efeito de cada trajetória nos valores de TFG estimada foram ajustados para as classes de peso ao nascer em relação à idade gestacional, o que confirmou que as diferenças de TFG encontradas eram independentes desse possível fator de confusão.

O principal ponto forte do nosso estudo é a inclusão de uma ampla amostra de crianças pré-púberes saudáveis, que se espera seja representativa da população em questão, o que nos leva a acreditar que nossos achados podem ser generalizados. De fato, apresentamos uma análise prospectiva de dados de uma *coorte* de base populacional, desde os primeiros anos de vida até a infância média, com dados sobre uma associação até então não descrita. As múltiplas medições de peso obtidas desde o nascimento permitiram uma análise do impacto na função renal, levando em consideração a “duração” e a “intensidade” do ganho de peso, bem como a idade de início. Além disso, utilizamos um modelo flexível ideal para identificar subpopulações com trajetórias longitudinais semelhantes. A análise dos resultados um ano após a última medição parece adequada em uma abordagem longitudinal. Adicionalmente, realizamos medições de cistatina C e creatinina em uma grande subamostra da *coorte*, com mais de 1.000 crianças entre 7 e 9 anos de idade, permitindo uma estimativa da TFG com base em ambos os marcadores, o que, como discutido anteriormente, apresenta vantagens na comparação com equações baseadas em um único marcador. A disponibilidade de informações detalhadas sobre inúmeras variáveis pré e pós-natais permitiu-nos considerar o impacto de potenciais fatores de confusão nas associações estudadas.

No entanto, é necessário reconhecer algumas limitações importantes de nosso estudo. A análise da função renal de forma transversal exige cautela nas interpretações de causalidade. Pretendemos superar essa limitação por meio de avaliações regulares dessas crianças, com particular interesse em seu estado nutricional e na evolução da sua função renal. Outra limitação importante é o fato de termos avaliado a função renal por meio do CrCl de 24 horas em 261 das 1.004 crianças incluídas no estudo. Isso reforça a necessidade de futuras coletas de dados para determinar melhor a natureza da associação entre as trajetórias de ganho de peso e a função renal, não só durante a infância, mas também futuramente, na idade adulta, e avaliar a possibilidade de um aumento da incidência de doença renal crônica e de menor sobrevida renal em adultos que, na infância, apresentaram um perfil de crescimento mais desfavorável. Outra limitação de nosso estudo é o fato de termos considerado somente uma medida geral (peso), e não a distribuição de gordura ou outros marcadores bioquímicos. As trajetórias de peso foram selecionadas, uma vez que estavam disponíveis medições repetidas de peso desde o nascimento até a primeira infância, permitindo um modelo longitudinal robusto. No entanto, o peso não distingue a massa gorda da massa magra nem a distribuição da gordura corporal. Por conseguinte, algumas trajetórias podem refletir, em parte, o crescimento somático constitucional, além da adiposidade. Estudos futuros que utilizem medidas diretas da composição corporal ou outros marcadores bioquímicos poderão permitir uma análise mais detalhada e ajudar a refinar essas associações. Entretanto, acreditamos que essa limitação foi amplamente superada pela quantidade de informação, com milhares de registros de peso ao longo do tempo, o que seria impossível de obter para outros marcadores.

Embora estatisticamente significativas, as diferenças na TFG estimada entre os grupos foram modestas em termos absolutos, e os valores permaneceram, em geral, dentro dos intervalos de referência pediátricos esperados. Nesse sentido, esses achados não devem ser interpretados como disfunção renal evidente, mas sim como possíveis diferenças fisiológicas ou subclínicas precoces que justificam um acompanhamento longitudinal. A coexistência de estimativas mais baixas de TFGe indexada e de maior CrCl absoluto em trajetórias de peso mais elevado pode refletir questões metodológicas relacionadas à normalização pelo tamanho corporal, hiperfiltração precoce ou ambas. Não estavam disponíveis marcadores adicionais de lesão renal, o que teria reforçado a interpretação global.

Nosso estudo nos levou à conclusão de que crianças com ganho de peso persistente de início precoce ou ganho de peso acelerado de início na infância apresentaram uma TFG significativamente mais baixa quando comparadas a crianças com ganho de peso mais lento e consistente. Esses achados podem refletir diferenças hemodinâmicas ou metodológicas precoces, em vez de disfunção renal estabelecida. Nossos resultados corroboram a influência dos padrões de crescimento na função renal, o que pode estar em consonância com outras associações anteriormente relatadas entre trajetórias de ganho de peso excessivo e um risco mais elevado de desenvolver obesidade e fatores de risco cardiovasculares em fases posteriores da vida^38^. Uma vez que as crianças incluídas em nosso estudo são muito jovens e ainda apresentam função renal dentro dos valores normais, mais estudos precisam ser realizados para estabelecer a relevância clínica de nossas conclusões. Como o início e o desenvolvimento da doença renal associada à obesidade são sutis e frequentemente permanecem não detectados durante anos, o uso generalizado de marcadores precoces — que já demonstraram bons resultados como métodos de triagem para lesão renal precoce em crianças com obesidade^4^ — poderá desempenhar um papel importante no futuro na detecção precoce e na prevenção de lesão renal nesse contexto.

Nossos achados destacam a importância da prevenção da obesidade desde a infância. Pesquisas futuras centradas na influência dos padrões de crescimento nos desfechos cardiovasculares e renais serão cruciais para auxiliar na identificação precoce de crianças em risco e para implementar intervenções oportunas que melhorem a saúde ao longo da vida.

## Data Availability

Os conjuntos de dados gerados e/ou analisados durante o presente estudo estão disponíveis junto ao autor correspondente, mediante solicitação razoável.
